# Objective Display and Discrimination of Floral Odors from *Amorphophallus titanum*, Bloomed on Different Dates and at Different Locations, Using an Electronic Nose

**DOI:** 10.3390/s120202152

**Published:** 2012-02-15

**Authors:** Kouki Fujioka, Mika Shirasu, Yoshinobu Manome, Nobuo Ito, Satoshi Kakishima, Tomohiro Minami, Tadashi Tominaga, Fumio Shimozono, Takeo Iwamoto, Keiichi Ikeda, Kenji Yamamoto, Jin Murata, Yasuko Tomizawa

**Affiliations:** 1 Department of Molecular Cell Biology, Institute of DNA Medicine, Jikei University School of Medicine, Tokyo 105-8461, Japan; E-Mails: kfujioka@jikei.ac.jp (K.F.); ikedak@jikei.ac.jp (K.I.); 2 Department of Applied Biological Chemistry, Graduate School of Agricultural and Life Sciences, The University of Tokyo, Tokyo 113-8657, Japan; E-Mail: mikashirasu@yahoo.co.jp; 3 Core Research Facilities, Jikei University School of Medicine, Tokyo 105-8461, Japan; E-Mail: tiwamoto@jikei.ac.jp; 4 Flower Park Kagoshima, Kagoshima 891-0513, Japan; E-Mails: n-itou@fp-k.org (N.I.); krp.minami@fp-k.org (T.M.); fpk@fp-k.org (T.T.); 5 Botanical Gardens, the University of Tokyo, Tokyo 112-0001, Japan; E-Mails: kakishima@ns.bg.s.u-tokyo.ac.jp (S.K.); murata@ns.bg.s.u-tokyo.ac.jp (J.M.); 6 Independent Researcher, Ibusuki, Kagoshima 891-0304, Japan; E-Mail: fumioshimonozo.ta@gmail.com; 7 National Center for Global Health and Medicine, Tokyo 162-8655, Japan; E-Mail: backen@ri.ncgm.go.jp; 8 Department of Cardiovascular Surgery, Tokyo Women’s Medical University, Tokyo 162-8666, Japan; E-Mail: stomizaw@hij.twmu.ac.jp

**Keywords:** electronic nose, FF-2A, semiconductor, *Amorphophallus titanum*, titan arum, smell, objective display, smell description

## Abstract

As olfactory perceptions vary from person to person, it is difficult to describe smells objectively. In contrast, electronic noses also detect smells with their sensors, but in addition describe those using electronic signals. Here we showed a virtual connection method between a human nose perceptions and electronic nose responses with the smell of standard gases. In this method, *Amorphophallus titanum* flowers, which emit a strong carrion smell, could objectively be described using an electronic nose, in a way resembling the skill of sommeliers. We could describe the flower smell to be close to that of a mixture of methyl mercaptan and propionic acid, by calculation of the dilution index from electronic resistances. In other words, the smell resembled that of “decayed cabbage, garlic and pungent sour” with possible descriptors. Additionally, we compared the smells of flowers which bloomed on different dates and at different locations and showed the similarity of odor intensities visually, in standard gas categories. We anticipate our assay to be a starting point for a perceptive connection between our noses and electronic noses.

## 1. Introduction

When we smell an object, chemical reactions occur between the odorous substances and high affinity olfactory receptors in the nose [1]. Although the scent of flowers is used to attract insects for the purpose of pollination [2], it is also one of the key factors in evoking human appreciation. A smell can be described as sweet, sour, or in any other manner depending on an individual’s perception [3]. However, it is difficult to objectively describe smells, apart from stating the chemical names of the odorants, as perceptions vary from person to person, with the human sense of smell also being influenced by learning, memory, emotion and language [4]. Thus measurements with an electronic nose should be useful to provide an objective evaluation of smells.

Studies involving electronic noses have been conducted for quality control of food and drink, odors associated with disease, chemicals, and detection of microorganisms [5–8]. The advantages of electronic noses are distinction and classification abilities. Recent studies showed reliable classification of beverages such as blackcurrant juice, mango juice and orange juice [9], and classification of honeys of different floral origin [10]. However, results obtained from electric noses are device-dependent, and it is difficult to compare results obtained from various devices or to compare with human perceptions. The reason for this disparity is that the sensors used in each device differ and olfactory receptors in humans differ from the sensors, in terms of number and sensing principle. To solve this problem, we configured our equipment such that it expresses the characteristics and intensity of smells in units of human perception, using an algorithm that incorporates the data of standard gases and human olfactory thresholds of the human nose [11].

Titan arum (*Amorphophallus titanum*), with the largest inflorescence in the world, is native to the tropical rainforests of Sumatra and is known to emit a strong carrion smell when flowering [12]. However, because it takes some 10 years from germination to flowering, there have been very few reports on the flowering of titan arum [13]. The carrion smell seems to attract not only insects but also researchers. Using gas chromatography mass spectrometry (GC–MS), Kite *et al.* showed that dimethyl oligosulfides (75% dimethyl disulfide [DMDS] and 10% dimethyl trisulfide [DMTS]) were the main chemical components of the carrion smell of the inflorescence of titan arum [14]. Most recently, Shirasu *et al.* identified DMTS as the main odorant that caused the rotting animal-like odor of the titan arum, by a GC–MS olfactometry technique using the human nose [15].

In this study, we evaluated and described the smell of two flowering titan arum samples (which are rarely cultivated outside their natural habitat and bloom only once every several years) to determine whether our measuring technique with an electronic nose and descriptor method, based on standard gases, is useful for the objective evaluation and description of the inflorescence smell.

## 2. Experimental Section

*Materials:* Nine diluted standard gases (methyl mercaptan, trimethylamine, heptane, propionic acid, butyl aldehyde, butyl acetate, toluene, ammonia, and hydrogen sulfide) were purchased from Sumitomo Seika Chemicals, Tokyo, Japan. Dimethyl disulphide and dimethyl trisulphide were also purchased from Wako Pure Chemical Industries, Osaka, Japan.

**Figure 1.**
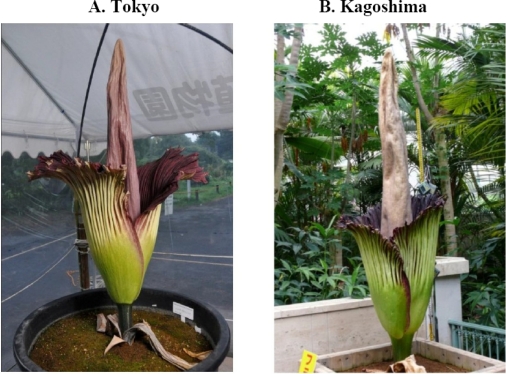
Titan arum grown in Tokyo (**A**) and Kagoshima (**B**), two locations in Japan those are approximately 1,000 km apart.

*Odor collection from Titan arum:* Titan arum plants grown at the Botanical Gardens, The University of Tokyo (Tokyo, Figure 1(a)) and the Flower Park Kagoshima (Kagoshima, Figure 1(b)) were studied. They were planted in 1993 using seeds provided by Dr. James R. Symon, an American botanist. The Tokyo odor sample was collected from the top of the spathe into a Tedlar bag (SUPELCO, Bellefonte, PA, USA) on 23 July 2010, and the Kagoshima sample was collected from around the appendix into a bag for odor sampling (Shimadzu Corporation, Kyoto, Japan) on 2 August 2010. Both samples were diluted fivefold in the sample bags using odorless dry nitrogen (>99.99995%, Japan Fine Products, Japan).

*Odor measurement and analyses:* Odor sample measurements were conducted using an FF-2A fragrance analyzer. This device is equipped with ten types of semiconductor sensors. By recording changes in the electrical resistance of the semiconductors when the chemical passes through the sensors, ten odor parameters can be recorded. The measuring principle and calculation method have been reported previously [11,16]. The electrical resistance values of three indicated concentrations of standard gases (hydrogen sulfide and ammonia; Table 1) for three sampling times (6 seconds, 18 seconds, and 60 seconds) from one indicated concentration of each standard gas (methyl mercaptan, trimethylamine, heptane, propionic acid, butyl aldehyde, butyl acetate, and toluene; Table 1) were recorded and used for drawing standard concentration-resistance curves. The sampling rate was 165 mL/min. After the resistances of flower samples were measured, the virtual concentration for each standard-gas axis was calculated by projecting the vector obtained to the axis. The dilution indexes were calculated with the division of virtual concentration by the concentration of human olfactory thresholds in each gas. In this study, the dilution indexes of five-fold concentrations of samples were calculated, since these samples were five-fold diluted with the dry nitrogen. The projecting and dilution indexes were calculated with Asmell 2 software (Shimadzu Corporation, Japan). The dendrogram analysis (average linkage) in this paper was produced using the statistical software SPSS Statistics 17.0 (IBM, USA).

## 3. Results and Discussion

**Figure 2.**
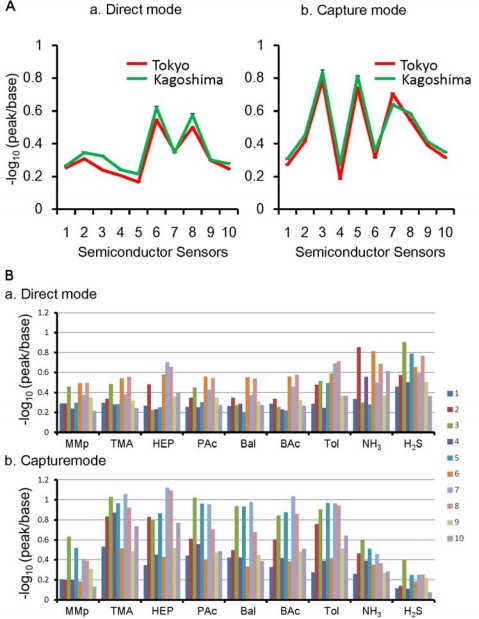
Measured values of odor samples. (**A**) inflorescences of titan arum plants at the 2 locations. To take into account the effect of humidity, measurements were conducted using the direct mode (a) and the capture mode (b); (**B**) Different response patterns of 10 semiconductors for each standard gas at one concentration. (a) direct mode; (b) capture mode. MMp; Methyl mercaptan (1 ppm), TMA; trimethylamine (1 ppm); HEP; heptane (3 ppm), Pac; propionic acid (2 ppm), BAL; butyl aldehyde (1 ppm), BAC; butyl acetate (1 ppm), Tol; toluene (3 ppm), NH_3_; ammonia (30 ppm), H_2_S; hydrogen sulfide (10 ppm).

Since 2003, the two titan arum plants used in this study have been grown separately in Tokyo and Kagoshima (Figure 1), two locations in Japan approximately 1,000 km apart. A FF-2A electronic nose was used to measure and record the odor samples. The values recorded by the ten sensors are shown in Figure 2(A).

In this study, to account for the effect of humidity on the samples, measurements were conducted both by the direct mode, which directly measures gases containing moisture (Figure 2(A)-a), and by the capture mode, which measures gases captured in a trap tube with moisture removed by heating (Figure 2(B)-b).

The former measures compounds with low boiling points and high water solubility, with the latter being suitable for compounds with high boiling points and poor water solubility. Using both methods doubles the odor pattern parameters. There were slight differences in values between the samples from Tokyo and Kagoshima, but the profiles of the two samples were similar in both modes, suggesting that the two samples had similar odor components.

In the present study, nine standard gases were incorporated into the algorithm (Table 1). These gases were selected with reference to the offensive odor substances as specified in the Japanese Offensive Odor Control Law. Metaphors for describing odors may vary from individual to individual, but by actually smelling standard gases under controlled conditions, it is possible to build a high level of consensus. Descriptors in the table are the odor descriptions in previous publications (Table 1) [17–20]. The results of the nine standard gases measured by the semiconductor sensors are shown in Figure 2B. The human nose identified odors based on the response patterns of their olfactory receptors, and the semiconductor sensors also showed a distinct reaction pattern for each standard gas.

**Figure 3.**
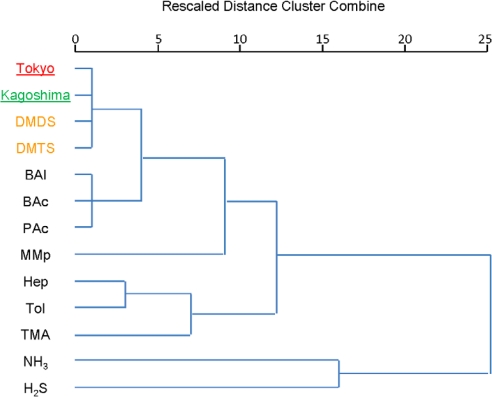
Cluster analysis (average linkage) of the discriminating power of the semiconductor sensors. A cluster analysis of titan arum odors from the 2 locations (Tokyo and Kagoshima), 9 standard gases and 2 principal components (0.01 ppm) was performed.

Using data from both modes, we determined the ability of semiconductor sensors to discriminate odors by cluster analysis (Figure 3). The results of this analysis showed that the Tokyo and Kagoshima samples of titan arum odors were more closely related to each other—and to DMDS, the principal component of titan arum odor as determined by GC–MS [14], as well as DMTS, the main odorant component of both samples perceived by the human nose [15]—than to any of the nine standard gases. This result suggested that these semiconductor sensors were suitable for differentiating the titan arum odor.

Next, the above data were analyzed with human olfactory thresholds incorporated into the analytical algorithm [11,16]. The vectors of the nine standard gases were constructed within the dimensions of ten sensors, and sample odors were positioned within the ten vectors, depending on the similarity with each gas. The odor samples were compared with standard gases taking into account not only the absolute concentrations but also the intensity of the odor as perceived by the human nose. This intensity is expressed as a “dilution index”, calculated by the algorithm incorporating the olfactory threshold (the dilution at which humans can no longer perceive any odor) of each standard gas. This concept is the same as that of the olfactometer method used for the measurement of odors present in the environment in Europe, and the odor index method employed in Japan.

**Figure 4.**
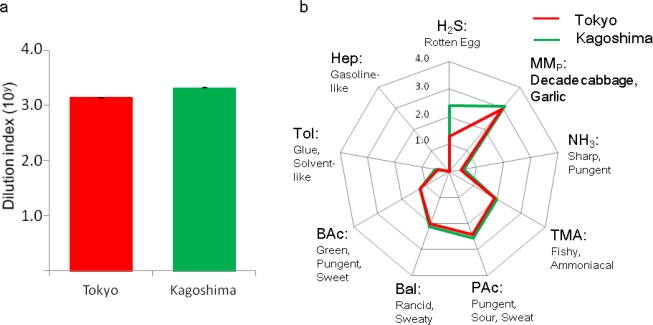
Odor display converted to levels of human perception by calculation and shown as dilution index. The overall intensity of the odor expressed in dilution index (10^y^) (**a**), and the intensities of odor components with respect to standard gas categories (odor contribution, (**b**) were shown (n = 3). For comparison of odor from the two locations, the Tokyo result in Figure 4(b) was quoted from a paper by Sirasu *et al*. [15].

When the overall intensity of the odor emitted by the titan arum was expressed as a dilution index, the intensity of the odor emitted by the Tokyo inflorescence was 10^3.2^ (1,585 times) and the Kagoshima inflorescence 10^3.3^ (1,995 times) (Figure 4(a), n = 3). The human nose cannot perceive odor intensity in a linear manner [21] and a difference of 10 times (one logarithm) or less may be perceived as roughly the same strength. The intensity of the odor emitted by the inflorescences in this study was almost equivalent to the intensity of the odor of kitchen waste [16].

Next, how did the inflorescences from the two locations smell, and was there a difference? The odor display, odor contribution diagram shows elongations in various axes, which suggested a mixture of different odors (Figure 4(b), n = 3). The intensity of the odor from titan arum was expressed with respect to the nine categories of offensive smelling standard gases. The compounds contributing to the odor from the inflorescence of the Tokyo plant have been reported by Shirasu *et al* [15]. In this study, we compared the odor contribution profiles of the Tokyo and the Kagoshima inflorescences, which bloomed on different days in different geographic locations, recalculated the odor levels relative to the human olfactory perception level and provided a perception of the odor, based on metaphors in previous publications [17–20] for standard gases (Table 1).

In both plants, the contribution by the sulfur category based on methyl mercaptan was high. This result indicates a smell resembling decayed cabbage and garlic [20]. The second highest contribution was the organic acid category based on propionic acid (pungent, sour and sweat) [17]. The third highest contribution was different between the two plants, with aldehydes (rancid and sweaty) [20] for the Tokyo inflorescence, and hydrogen sulfide for the Kagoshima inflorescence (rotten eggs) [20]. This difference may be due to sample humidity, because the Tokyo plant was placed in open air whereas the Kagoshima was placed in a conservatory when they bloomed. Therefore, the estimated intensity of hydrophilic odor resembling hydrogen sulfide may be higher with the Kagoshima plant. The other categories were difficult to perceive with the human nose without paying special attention, because the odor contribution levels were below 10^2^ and the intensities were more than ten times lower than the three main categories.

In previous publications, “carrion smell” was used to describe the smell of the titan arum inflorescence, but by using metaphors for standard gases to express the overall odor of titan arum, we can describe it as a mixture of methyl mercaptan and propionic acid; in other words, its smell resembles that of “decayed cabbage, garlic and pungent sour”. The odor of DMTS, the main odorant component detected by the human nose [15], was described as “garlic and gas” in another report [22]. This description is compatible with the metaphor by our electronic nose and descriptors, based on the standard gases method. Although odor descriptors are known to be based on their cultural background [23,24], we can share odor images among different cultures, through the intermediation of the odor of standard gases.

It is notable that the odor contributions for the Tokyo [15] and Kagoshima inflorescences overlap almost completely except for the hydrogen sulfide category. This result from objective analysis showed that for titan arum blooming on different dates and locations, not only the overall odor intensity but also the individual odor components are essentially identical.

## 4. Conclusions

In this study, we examined a method for objectively comparing and describing odors with semiconductor sensors and nine standard gases. We showed that, using an electronic nose and standard gas descriptors, the carrion smell of *Amorphophallus titanum* flowers could be objectively described as “decayed cabbage, garlic and pungent sour.” This description was compatible with the metaphor description of DMTS, the main odor component of the flower detected by the human nose. Additionally, we visually displayed the similarity of odor from the flowers which bloomed on different dates and at different locations.

Our results suggested that smells at different times and locations could be recorded objectively, with respect to standard gas categories, and these recoded odors have the potential to convey the human-perceived smells to people. Using this method, it should be possible to compare odors today and in 10 years’ time and for different countries or places. The method cannot identify components like gas chromatography can, but it can express odors in an easily understandable manner with electronic noses and the information can be shared across time and space.

However, it is difficult to explain pleasant smells, such as roses or citruses, using the malodorous standard gases used in this study. To further develop this research, we need to increase the number of standards that have metaphors exhibiting high international and cross-cultural consensus. The strategy is analogous to that of sommeliers who use familiar smells and tastes to convey a clear description of wines. By classifying odors into familiar categories and quantifying them, it will be easier to evaluate and compare odors. This may ultimately perceptualize smell objectively and find wide applicability.

## Figures and Tables

**Table 1.** Standard gases and metaphor examples.

**Smell Group**	**Standard gas (Abbreviation)**	**Concentration (ppm)**	**Possible descriptor & Reference number**
Hydrogen sulfide	Hydrogen sulfide (H_2_S)	10, 3, 1	Rotten eggs [12]
Sulfur	Methyl mercaptan (MMp)	1	Decayed cabbage, garlic [12]
Ammonia	Ammonia (NH_3_)	30, 10, 3	Sharp, pungent [12]
Amine	Trimethylamine (TMA)	1	Fishy, ammoniacal [12]
Organic acid	Propionic acid (PAc)	2	Pungent, sour, sweat [13]
Aldehyde	Butyraldehyde (Bal)	1	Rancid, sweaty [12]
Ester	Butyl acetate (BAc)	1	Green, pungent, sweet [14]
Aromatic group	Toluene (Tol)	3	Glue, solvent-like [14]
Carbon hydrate	Heptane (Hep)	3	Gasoline-like [15]
